# Efficacy of different cavity liners compared to no cavity lining in managing deep caries in vital permanent teeth - a systematic review

**DOI:** 10.1007/s00784-025-06479-y

**Published:** 2025-10-24

**Authors:** Esra Kosan, Deniz Kosan, Richard Sturm, Falk Schwendicke, Sebastian Paris

**Affiliations:** 1 Department of Periodontology, Oral Medicine and Oral Surgery, Charité - Universitaetsmedizin Berlin, Berlin, Germany; 2 Medical Student, Medical School Berlin (MSB), Berlin, Germany; 3 Department of Operative and Preventive Dentistry, Charité - Universitaetsmedizin Berlin, Berlin, Germany; 4 Department of Conservative Dentistry and Periodontology, Ludwig-Maximilians-University of Munich, Munich, Germany

**Keywords:** Dental caries, Dental cavity lining, Vital pulp therapy, Restorative dentistry, Systematic review

## Abstract

**Objective:**

This review aimed to evaluate whether the use of a certain cavity lining material (I) in patients with deep caries lesions in mature or immature permanent teeth associated with no symptoms or reversible pulpitis (P) is as efficacious as other materials or no cavity lining (C) in terms of patient-reported and clinically-reported outcomes (O), with “failure” identified as the primary critical outcome.

**Materials and methods:**

Two independent reviewers selected studies, extracted data, and assessed the risk of bias. The systematic literature search was restricted to English-language publications using Cochrane Review, PubMed (Medline) and Ovid databases. Search strategies included combinations of free keywords, controlled vocabulary terms (Medical Subject Headings-MeSH), Boolean operators, truncations and proximity operators. Eligible studies included randomized controlled trials (RCTs), and comparative clinical trials (CCTs). Due to variability in reported outcomes, a meta-analysis was not conducted. The quality of evidence was assessed using the Grading of Recommendations, Assessment, Development and Evaluations (GRADE) approach.

**Results:**

Analysis of 12 studies showed no significant differences in clinical outcomes between restorations with and without cavity liners. Studies directly comparing restorations with and without liners showed no statistically significant differences in key outcomes such as failure rates, postoperative hypersensitivity or secondary caries. (GRADE: low certainty) and minimizing postoperative hypersensitivity (GRADE: very low certainty). The effect of cavity lining on tooth survival (success rate: 94%-100%) and restoration longevity (failure rate: 0%-6%) was of low certainty.

**Conclusion:**

Cavity liners, regardless of the material, do not consistently deliver superior clinical outcomes compared to no liners.

**Clinical relevance:**

The findings of this systematic review suggest that routine use of cavity liners in deep caries management may not be necessary for achieving successful restorative outcomes.

## Introduction

Dental caries is one of the most widespread diseases and continues to be a significant global health burden. In advanced stages, beside the dental hard tissues, caries often also affects the pulp. When treating these stages restoratively, it is essential to protect the pulp tissue to preserve the tooth vitality and avoid further complications [1–3]. Cavity liners have traditionally been employed in such cases to protect the pulp from thermal, chemical and mechanical stimuli, as well as to enhance patient comfort. However, their clinical efficacy compared to no liner application remains a subject of debate [1].

Minimally invasive approaches, broadly grouped under the term Vital Pulp Therapies (VPT), have gained traction as conservative options for managing deep carious lesions. Unlike conventional methods such as root canal treatment or full pulpectomy, VPT is intended to preserve the health and function of the remaining pulp tissue, stimulate healing and encourage the formation of reparative dentin bridges [2–4]. Various biomaterials, including calcium hydroxide formulations, mineral trioxide aggregate (MTA) and more recent hydraulic calcium silicate cements, are advocated in this context for their purported ability to foster pulpal repair, seal exposed areas against microleakage and diminish postoperative discomfort [5–7]. Some materials are also claimed to improve the sealing of microgaps and strengthen adhesion of the final restoration [8]. Despite these theoretical advantages, their long-term clinical benefits remain contested, particularly in terms of pulp preservation, reduction of postoperative hypersensitivity and prevention of secondary caries [3].

A recent Cochrane systematic review [9] compared the use of dental cavity liners to no liners for Class I and Class II resin-based composite restorations. The review highlighted the limited and inconsistent quality of available evidence, casting doubt on the routine use of liners in deep caries management. Traditional materials like calcium hydroxide were questioned for their necessity, while alternatives such as glass-ionomer cement (GIC) or adhesive bonding systems were noted for comparable or better performance in certain contexts. Moreover, although resin-modified calcium silicate-based cements have shown promise in indirect pulp capping, their outcomes in direct pulp capping remain less predictable [3].

This systematic review was undertaken as part of an S3-level evidence-based guideline initiative supported by leading professional organizations, including the European Federation of Conservative Dentistry (EFCD), the European Society of Endodontology (ESE), the European Organization for Caries Research (ORCA), and the German Society for Restorative Dentistry (DGZ). The primary aim was to evaluate the efficacy of different cavity liner materials (e.g., glass-ionomer cement, calcium hydroxide or silicate-based materials) compared to no liner use in managing deep caries in permanent teeth. Outcomes assessed included pulp and tooth survival, secondary caries and patient-reported symptoms, with the goal of providing clinically relevant insights into the necessity of liners in contemporary restorative practice.

## Methods

### Protocol and registration

This systematic review adhered to the Preferred Reporting Items for Systematic Reviews and Meta-Analyses (PRISMA) standards. A detailed protocol outlining the objectives and methodology was registered prospectively with the International Prospective Register of Systematic Reviews (PROSPERO; registration number: CRD42023446052).

### Original review question and subsequent modification

The original question applied the PICO framework:

P (Population): Patients with deep caries lesions in mature or immature permanent posterior teeth (Class I and II), with no symptoms or reversible pulpitis.

I (Intervention): Use of a specific cavity liner.

C (Comparison): No liner.

O (Outcome): Patient- and clinically reported outcomes; primary: tooth survival.

T (Time):

- Tooth survival and other clinical outcomes: ≥12 months.
- Pain-related outcomes: 7 days to 3 months.
- OHRQoL: ≥6 months.

S (Study design): Randomized Controlled Trials (RCTs), Comparative Clinical Trials (CCTs).

During the initial scoping phase, we observed a scarcity of direct comparative studies evaluating cavity liners versus no liners. However, several trials compared different types of liners (e.g., calcium hydroxide vs. MTA, or glass-ionomer cement vs. TheraCal). To ensure a broader and more representative analysis of the existing evidence, we expanded the review question to include studies comparing one liner material to another, in addition to those comparing liners to no liners. This modification did not change the inclusion criteria regarding population, study design (RCTs, CCTs), or core outcomes. Instead, it allowed for the inclusion of more studies while preserving methodological consistency.

For analytical clarity, the studies were grouped into two categories: those comparing a cavity liner with no liner (Group A) and those comparing one type of cavity liner with another (Group B). The findings from each group were synthesized narratively, with thematic summaries provided separately in the Results section to maintain clarity and structure. Findings from these subgroups were synthesized narratively, with thematic summaries provided separately in the Results section to maintain clarity.

### Final review questions

Accordingly, the review question was refined to:

In patients with deep caries lesions in mature or immature permanent teeth associated with no symptoms or those of a reversible pulpitis (P), is a cavity lining of a certain material (I) as efficacious as other materials or no cavity lining (C), in terms of a combination of patient and clinical reported outcomes (O), with “failure” as the most critical outcome?

### Definitions

The classification of pulpal health was derived from the American Association of Endodontists consensus recommendations (2009) [10], with categories defined as follows:

- *Normal pulp*: No clinical symptoms and normal responses to vitality tests.
- *Reversible pulpitis*: Discomfort that arises solely in response to external stimuli and subsides shortly after the stimulus is removed.
- *Symptomatic irreversible pulpitis*: Spontaneous pain episodes or prolonged sensitivity (lasting over 30 s) following stimulation, frequently necessitating endodontic intervention.

#### Definition of failure

Treatment failure was determined if the tooth demonstrated loss of pulp vitality requiring further procedures (e.g., pulpectomy), if the restoration deteriorated (fractured, debonded, or was lost) prompting repair or replacement, or if the tooth required extraction due to persistent pathology. Failures were assessed using clinical indicators (such as sensitivity and pain), radiographic signs (such as periapical radiolucency or resorption) or the need for medication. Failure was assessed over specific follow-up periods tailored to the nature of each outcome. Clinical and radiological outcomes, including tooth survival and restoration integrity, required a minimum follow-up of 12 months to ensure meaningful evaluation. Pain, tenderness, swelling, and the need for analgesics were monitored over a shorter period, ranging from 7 days to 3 months, to capture immediate post-treatment responses. For oral health-related quality of life (OHRQoL), a minimum follow-up of six months was necessary to assess sustained patient-centered outcomes.

### Eligibility criteria

#### Inclusion criteria

Studies (RCTs and CCTs) were included if they involved permanent teeth (either mature or immature) with deep caries and a diagnosis of reversible pulpitis. Eligible interventions included any form of cavity liner applied before final restoration. Comparators could be either no liner or a different liner material. The required follow-up durations were 12 months or more for clinical outcomes, 7 days to 3 months for pain and medication use and 6 months or more for OHRQoL.

#### Exclusion criteria

Studies were excluded if they were non-comparative, focused solely on primary teeth, were in vitro or animal studies, were not published in English, lacked results (e.g., registered but incomplete trials), or were case reports, case series, or any form of review (narrative, scoping, or systematic). The language restriction was due to feasibility and the need for precise interpretation of clinical terms, which was not possible for non-English studies within the scope of this review.

### Search strategy

An electronic literature search limited to English-language studies was conducted between 30 August and 30 October 2023, with an update between 1 December 2024 and 1 February 2025. Databases included the Cochrane Central Register of Controlled Trials (CENTRAL), PubMed (including MEDLINE), and Ovid. The search approach incorporated a combination of free-text terms, standardized MeSH descriptors, Boolean logic connectors, truncation techniques and proximity searching to identify all potentially relevant records. The strategy was developed in collaboration with an information specialist and a librarian. Additional manual searches were performed in key journals (e.g., International Endodontic Journal, Journal of Endodontics, Journal of Dental Research, Journal of Dentistry and Clinical Oral Investigations). Grey literature sources (OpenGrey, Google Scholar, Open Access Theses and Dissertations) were also screened. Scopus was not searched due to significant database overlap but may be considered in future updates.

Detailed search strategies are provided in Appendix Table 5.

### Study selection

Study selection was carried out in two phases. Titles and abstracts from retrieved documents were assessed independently and in duplicate by two reviewers (EK, RS) who were not blinded. However, to minimize selection and extraction bias, the reviewers underwent calibration sessions prior to screening and data extraction. During these sessions, they jointly reviewed a subset of studies to ensure consistency in applying inclusion criteria and data recording procedures. Papers not meeting the inclusion criteria were excluded with reasons (Table 1) and full texts of initially selected articles were sources for further evaluation. Disagreements and doubts were resolved by discussion with a third reviewer. The search was rerun before conducting the final analyses and newly found eligible texts was included.

**Table 1:** Excluded studies and reasons for exclusion

Excluded studies	Reasons for exclusion
Bjørndal et al. 2010 [11]	Wrong Comparator
Corralo and Maltz 2013 [12]	Wrong Outcome
Marending et al. 2016 [13]	Wrong Study Design
Brännström et al. 1991 [14]	Wrong Study Design
Ruiz and Mitra 2006 [15]	Wrong Study Design
Weiner 2011 [16]	Wrong Study Design
Weiner 2008 [17]	Wrong Study Design
Weiner 2002 [18]	Wrong Study Design
Hilton 1996 [19]	Wrong Study Design
Cox and Suzuki 1994 [20]	Wrong Study Design
Akpata and Sadiq 2001 [21]	Wrong Time Frame (Fllow-up shorter than 12 m)
Alqahtani et al. 2020 [22]	Wrong Time Frame (Fllow-up shorter than 12 m)
Burrow et al. 2009 [23]	Wrong Time Frame (Fllow-up shorter than 12 m)
Kaurani and Bhagwat 2007 [24]	Wrong Time Frame (Fllow-up shorter than 12 m)
Strober et al. 2013 [25]	Wrong Time Frame (Fllow-up shorter than 12 m)
Wegehaupt et al. 2009 [26]	Wrong Time Frame (Fllow-up shorter than 12 m)
“Clinical and Radiographic Comparison of Biodentine and Calcium Hydroxide Cement as Indirect Pulp Capping Agents.” [27]	Protocols only – no Results
“Comparison and Evaluation Of success of tricalcium silicate-based filling material, light-curable calcium hydroxide, and mineral trioxide aggregate as indirect pulp capping filling materials between deciduous (milk teeth) and permanent teeth.” [28]	Protocols only – no Results

### Data extraction

Two reviewers (EK and DK) independently extracted data using a piloted form. Any discrepancies were resolved through discussion or, if needed, consultation with a third reviewer (RS). For studies with multiple arms or duplicate publications, only relevant data were included. In case of incomplete or missing data, the authors of the papers were contacted for clarification.

Extracted fields included:

- Author/year.
- Participants/dropouts.
- Intervention type.
- Outcomes assessed (clinical and patient-reported).
- Radiographic findings.
- OHRQoL metrics.

Data are presented in Appendix Table 4.

### Risk of bias assessment

Two independent authors (EK, DK) graded the risk of bias using the Cochrane Risk of Bias tool (RoB) which covered the domains of sequence generation, concealment of allocation, participant and personnel blinding, completeness and transparency of outcome reporting, and the handling of incomplete data [8]. The overall risk of bias was classified in three categories:

(1) Low risk of bias - The study is well-conducted with minimal concerns about bias.
(2) Some concerns - There is uncertainty due to missing information or methodological weaknesses that may introduce bias.
(3) High risk of bias -The study has significant flaws that are likely to distort the results.

Other instruments were not applied since only RCTs were included in this review. Any disagreements between the reviewers regarding the risk of bias were resolved through discussion, and, if necessary, a third reviewer (RS) was consulted.

### Grading of evidence

The overall strength of evidence was appraised following the GRADE framework (Grading of Recommendations, Assessment, Development and Evaluation), emphasizing the primary outcome of treatment failure. Two reviewers (EK and DK) independently rated each domain and resolved discrepancies through discussion until consensus was reached. Prior to the evaluation, they underwent a calibration exercise to ensure consistent interpretation of GRADE criteria. Justifications for downgrading or upgrading the certainty of evidence (e.g., risk of bias, inconsistency, imprecision) are presented in Table 2.

**Table 2:** Quality assessment for tooth survival, postoperative hypersensitivity, secondary caries, and restoration longevity using GRADE

Outcome	Number of studies	Number of treated teeth (drop-out rate)	Study limitation (Risk of Bias)	Inconsistency of results	Indirectness of evidence	Imprecision	Publication bias	Participants age	Effect	Quality of evidence
Failure	12 (RCT) [29–35, 37, 39, 40]	1184 (~ 14.1%)	Serious limitations	Serious limitations	Serious limitations	Serious limitations	NA	6–76 years (4/12 studies included children)	No significant difference; 96.5% of treated teeth remained vital	⊕○○○ (Very Low)
Tooth Survival	12 (RCT) [29–40]	1184 (~ 14.1%)	Serious limitations	Serious limitations	No limitations	Serious limitations	NA	6–76 years (4/12 studies included children)	No significant difference; 99.4% survival in all groups	⊕⊕○ ○ (Low)
Postoperative Hypersensitivity	9 (RCT) [29–31, 35–40]	1000 (~ 12.1%)	Very Serious limitations	Very Serious limitations	Serious limitations	Very Serious limitations	NA	6–54 years (2/9 studies included children)	No significant difference; minimal pain reported	⊕○○○ (Very Low)
Secondary Caries Prevention	10 (RCT) [29, 30, 32, 34–38, 40]	1009 (~ 11.7%)	Serious limitations	Very Serious limitations	Serious limitations	Very Serious limitations	NA	6–54 years (4/10 studies included children)	No significant difference; ~100% prevention	⊕⊕○○ (Low)
Restoration Longevity	8 (RCT) [29, 30, 32, 34, 36–38, 40]	889 (~ 12.7%)	Serious limitations	Serious limitations	No limitations	Serious limitations	NA	6–54 years (3/8 studies included children)	No significant difference; high success in all groups	⊕⊕○ ○ (Low)

Indirectness of Evidence was downgraded for the outcomes “Secondary Caries Prevention” and “Postoperative Hypersensitivity” due to indirectness, as some studies used alternative liner materials or did not directly compare cavity liners to no liners, reducing direct applicability to the research question

Study Limitations (Risk of Bias) was downgraded due to serious limitations caused by randomization and allocation concealment issues, increasing the risk of selection bias. Blinding of assessors was inconsistently reported, contributing to potential detection bias. However, operator blinding was not considered a limitation, as it is typically impractical in restorative procedures

Inconsistency was downgraded due to serious heterogeneity caused by variations in liner materials, caries removal techniques, and follow-up durations. This contributed to inconsistent findings, particularly for the outcomes “Restoration Longevity” and “Postoperative Hypersensitivity”, where different measurement tools and evaluation criteria were used

Imprecision was downgraded due to small sample sizes and relatively short follow-up durations, reducing statistical power and increasing uncertainty in long-term clinical outcomes. This was particularly relevant for restoration longevity, secondary caries prevention, and postoperative hypersensitivity, where long-term effects remain unclear

There was no strong evidence of publication bias, but the limited number of large, high-quality RCTs suggests a potential for selective reporting. The lack of long-term data further limits the robustness of conclusions

GRADE rates the quality of evidence into four levels:

⊕⊕⊕⊕ (High): Further research is very unlikely to change our confidence in the estimate of effect.

⊕⊕⊕○ (Moderate): Further research is likely to have an important impact on our confidence in the estimate of effect and may change the estimate.

⊕⊕○○ (Low): Further research is very likely to have an important impact on our confidence in the estimate of effect and is likely to change the estimate.

⊕○○○ (Very Low): Any estimate of effect is very uncertain.

### Data synthesis strategy

All data was analysed qualitatively and quantitatively, and a narrative synthesis of the included studies was performed. Since the included studies were not homogeneous in nature, a quantitative meta-analysis was not performed.

Where available, exact p-values are reported to enhance transparency and interpretability of findings. In cases where original authors only stated non-significance (e.g., “p > 0.05”) or omitted p-values entirely, this has been indicated accordingly.

## Results

### Study selection

**Fig. 1:**
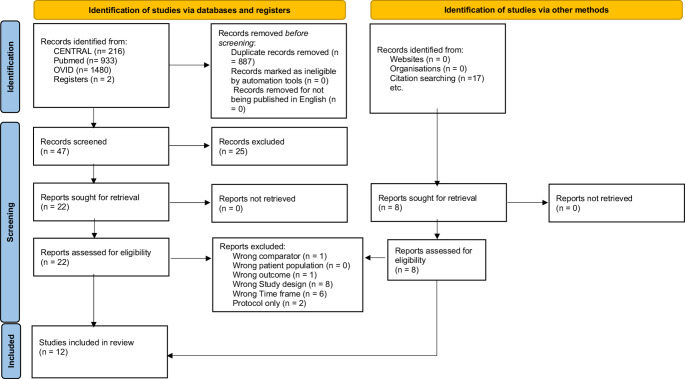
PRISMA 2020 flow diagram for new systematic reviews which included searches of databases, registers and other sources

The literature search is summarised in the PRISMA flowchart in Fig. 1. A total of 934 records were initially identified through database and manual searches. After removing duplicates, 761 records remained for title and abstract screening. Of these, 714 were excluded for not meeting the inclusion criteria. Forty-seven full-text articles were then assessed for eligibility. Following full-text evaluation, 18 studies were excluded for the following reasons: wrong comparator (*n* = 1 [11]), wrong outcome (*n* = 1 [12]), unsuitable study design (*n* = 8 [13–20]), inadequate follow-up duration (*n* = 6 [21–26]), and incomplete trials (*n* = 2 [27, 28]). Ultimately, 12 articles were included in the qualitative synthesis (Table 1).

### Study characteristics and outcome assessment

A summary of the studies included in this systematic review is presented in Appendix, Table 4.

The included studies demonstrated considerable variation in participant characteristics, outcomes assessment tools and methodological rigor. Participant ages ranged from children (as young as 6 years) to adults (up to 76 years), with a mix of male and female participants the male-to-female ratio varied across studies, though it was generally balanced. Outcomes were assessed using diverse tools, including clinical indices (e.g., FDI criteria, modified USPHS criteria), patient-reported measures (e.g., VAS for pain) and pulp vitality tests (e.g., cold and electric pulp testing). While some studies, such as Singh et al., Banomyong et al. Bayoumy et al., Ahmed et al., Pereira et al., Hashem et al. and Gürcan and Seymen [29–35], conducted robust sample size calculations to ensure adequate statistical power, others lacked such calculations or did not report them. Inclusion criteria typically focused on deep carious lesions in permanent teeth with vital pulps, while exclusion criteria often eliminated patients with severe periodontal issues, systemic diseases or teeth with irreversible pulpitis or nonvital pulps. Recall periods for outcome assessments varied: clinical outcomes such as restoration failure, pulp vitality and secondary caries were primarily evaluated at 12 to 36 months. Postoperative pain and hypersensitivity were typically assessed within 7 days to 3 months.

Across the included studies, cavity liners investigated included calcium hydroxide-based materials (e.g., Dycal) [31, 32, 35], resin-modified calcium silicates (e.g., TheraCal LC) [31, 32], glass-ionomer cements [30, 34, 36, 37], hydraulic calcium silcate cements (Biodentine or MTA) [32, 33] and flowable composite liners [38]. In addition, less conventional materials were assessed, including a resin-based ion-releasing liner (Activa BioACTIVE) [29], a desensitizing polycarboxylate cement containing potassium nitrate [39] and silver diamine fluoride (SDF) used as a pre-treatment prior to liner application [40]. Comparator groups used either alternative liner types [29, 31–33, 39, 40] or no liner [30, 34–38]. Interventions were applied in diverse restorative contexts involving composite resin [29–32, 34–38, 40], amalgam [39], or glass-ionomer restorations [33].

### Primary outcome: failure

Failure was commonly defined across studies as the loss of pulp vitality requiring endodontic intervention (pulpotomy, pulpectomy), restorative retreatment due to restoration loss or fracture or tooth extraction. Most studies evaluated pulp vitality using cold and/or electric pulp testing, though the specific protocols and interpretation thresholds were not always clearly reported [33–35]. Radiographic assessments to identify periapical changes or resorptive processes were included in some studies [31, 33, 34], while others [30, 32] did not report radiographic follow-up or used it inconsistently. Standardized clinical criteria were also applied to assess restoration success and integrity, like the FDI criteria [29, 37] and the modified USPHS criteria [32]. Recall intervals also differed; while most studies reported outcomes only at a final follow-up point, typically at 12 or 24 months, some [29] included multiple follow-ups over an extended period, with outcomes reported at 6, 12, and 36 months.

Gürcan and Seymen [32], rated as high risk of bias due to unclear randomization and outcome assessment methods, reported restoration success rates of 84.6–94.4% across Dycal, ProRoot MTA, and TheraCal groups, with no statistically significant differences (*p* > 0.05). Hashem et al. [33], also assessed as high risk of bias, found identical pulp vitality rates of 83.3% in both the Biodentine and Fuji IX (GIC) groups, again with no significant difference in failure rates (*p* = 0.91). Bayoumy et al. [31], with a moderate risk of bias due to unclear allocation concealment and absence of radiographic outcomes, observed restoration success rates of 94.7% for TheraCal and 89.5% for Dycal, but reported no statistically significant difference in failure (*p* > 0.05). Ahmed et al. [29], also rated as having moderate risk of bias, found comparable long-term success between so called “ionic liners” and resin-modified GICs over 36 months; however, p-values were not reported, and outcome details were limited. Torres et al. [37], with a moderate risk of bias, found failure rates below 5% in all groups, regardless of liner use, and observed no significant impact on restoration longevity (*p* > 0.05). Pereira et al. [34], also rated as moderate, reported a pulp vitality rate of 98.6% with no added benefit from liner use (*p* > 0.05). Among studies with low risk of bias, Banomyong et al. [30] and Singh et al. [35] both reported no significant differences in failure rates between groups using different liners or no liner at all. Singh et al. observed pulp survival rates exceeding 94% across calcium hydroxide, GIC, and unlined groups (*p* = 0.71). Banomyong et al. and Banomyong and Messer [36] (the latter rated with moderate risk of bias) also reported low failure rates for both lined and unlined restorations (*p* > 0.05).

### Secondary outcome: patient reported outcomes

Patient-reported outcomes were primarily assessed through measures of postoperative pain or hypersensitivity (POHS), with limited reporting on functional satisfaction or long-term comfort. The majority of studies found that the use of cavity liners did not significantly enhance patient comfort.

Banomyong et al. [30], rated low risk of bias, and Banomyong and Messer [36], moderate risk of bias, reported no significant differences in postoperative hypersensitivity between restorations with and without liners (*p* > 0.05), indicating that liner application did not improve patient comfort. Similarly, Pereira et al. [34] and Torres et al. [37], both with moderate risk of bias, found low pain and sensitivity levels across all groups, with no statistically significant differences based on liner application (*p* = 0.402 and *p* > 0.05, respectively). In contrast, Bayoumy et al. [31] reported a reduction in postoperative sensitivity with TheraCal, a resin-modified calcium silicate liner, compared to Dycal, with 100% of patients in the TheraCal group reporting no pain at 12 months versus 81% in the Dycal group (*p* < 0.05). However, the study’s moderate risk of bias, due to unclear allocation concealment and lack of radiographic follow-up, limits the certainty of this finding. Similarly, Hodosh et al. [39] observed a significant reduction in postoperative hypersensitivity with the use of potassium nitrate liners (*p* < 0.05), while having a low risk of bias.

Economic aspects were not explicitly analyzed in the included studies.

### Subgroup analysis

#### Cavity liner vs. no cavity liner

Multiple randomized controlled trials found no significant differences in clinical outcomes—such as failure rates or postoperative hypersensitivity—between restorations with and without cavity liners. High-quality studies by Singh et al. [32] and Banomyong et al. [30] (both low risk of bias) support the conclusion that liners are not clinically essential. Other studies, including Banomyong and Messer [36], Torres et al. [37], Pereira et al. [34], and Efes et al., also found no benefit from liner use, though their moderate risk of bias warrants more cautious interpretation.

#### Cavity liners of various material

While cavity liner materials such as CH, GIC, TheraCal, and MTA differ in composition and handling, studies generally show no significant differences in long-term clinical outcomes [30, 33–40]. Some materials marketed as “bioactive” were evaluated as well [29, 31, 32]. The term “bioactive ionic liners,” as used in this review, refers specifically to resin-based liner materials such as TheraCal LC and Activa BioACTIVE that are designed to release therapeutic ions (e.g., calcium, phosphate and fluoride) stimulating remineralization and dentin bridge formation while maintaining physical properties suitable for light-curing and use under restorations [29]. Unlike traditional calcium hydroxide or glass- ionomer cements, these liners incorporate “bioactive” glass or calcium silicate components within a resin matrix. In Ahmed et al. [29] outcomes were similar between an ion-releasing resin-based liner and a traditional RMGIC, while Bayoumy et al. [31] reported significantly (*p* < 0.05) reduced POHS with TheraCal compared to Dycal, though the study was rated with moderate risk of bias. Gürcan and Seymen also compared TheraCal to Dycal and MTA, revealing high success rates for all materials (no statistically significant differences), but the high risk of bias limits the reliability of these findings. Hodosh et al. [39] used a potassium nitrate-polycarboxylate cement liner and compared it to a polycarboxylate cement. They observed a statistically significant reduction of POHS (*p* < 0.01) in the potassium nitrate group. Baraka et al. [40] compared the effect of SDF application prior to restoration with GIC, not the effect of different liners. SDF reduced POHS (though not statistically significant), but the finding does not support the efficacy of one liner material over another.

Overall, the body of evidence, including all 12 trials, does not support the superiority of any specific liner material.

#### Risk of bias

All included studies were analysed for risk of bias, with varying levels of methodological quality. Table 3 outlines the details of the risk of bias.

**Table 3:** Risk of bias assessment using the ROB tool

STUDY ID	D1	D2	D3	D4	D5	OVERALL RISK OF BIAS
Baraka et al., 2022	Low	Low	High^1^	Low	Low	**Some Concerns**
Efes et al., 2006	Some concerns^2^	Low	Low	Some concerns ^2^	Low	**Some Concerns**
Banomyong et al., 2011	Low	Low	Low	Low	Low	**Low**
Torres et al., 2020	Low	Low	High^3^	Some concerns^3^	Low	**Some Concerns**
Ahmed et al., 2024	Low	Low	Low	Low	Low	**Low**
Banomyong and Messer, 2013	Some concerns^4^	Low	Low	Some concerns^4^	Low	**Some Concerns**
Pereira et al., 2017	Low	Low	Some concerns^5^	Low	Low	**Some Concerns**
Hodosh et al., 1991	Low	Low	Low	Low	Low	**Low**
Bayoumy et al., 2021	Low	Low	Low	Some concerns^6^	Low	**Some Concerns**
Singh et al., 2019	Low	Low	Low	Low	Low	**Low**
Gürcan and Seymen, 2019	High^7^	High^7^	High^7^	High^7^	Low	**High**
Hashem et al., 2015	High^8^	High^8^	High^8^	High^8^	Low	**High**
Bayoumy et al., 2021	Low	Low	Low	Low	Low	**Some Concerns**

Domains:

D1: Bias arising from the randomization process

D2: Bias due to deviations from intended intervention

D3: Bias due to missing outcome data

D4: Bias in measurement of the outcome

D5: Bias in selection of the reported result

^1^ High loss to follow-up

^2^ Unclear sequence generation, lack of postoperative radiographic diagnosis and no sample size calculation

^3^ Incomplete outcome data and selective reporting

^4^ Unclear sequence generation and lack of postoperative radiographic diagnosis

^5,6^ Unclear blinding of outcome assessment

^7^Randomization process not described, and allocation concealment was unclear; no blinding of operator; missing outcome data not reported or addressed; outcomes assessed by the treating operator without blinding

^8^ No clear description of random sequence generation or allocation concealment, no information on blinding of participants or personnel; missing outcome data not properly addressed or explained, unclear blinding of outcome assessment

Several studies, including Singh et al. [35], Hodosh et al. [39], Ahmed et al. [29] and Banomyong et al. [30] were rated as having a low risk of bias, as they scored “Low” in key domains such as randomization, allocation concealment, blinding of outcome assessment, and follow-up. However, two studies, Gürcan and Seymen [32] and Hashem et al. [33], revealed a high risk of bias due to unclear reporting of randomization procedures and lack of blinding. Although blinding of operators and evaluators was not feasible in some trials due to the nature of the intervention, studies that implemented objective outcome assessments and complete follow-up were considered to have a lower overall risk of bias.

### Meta-analysis

A meta-analysis was deemed inappropriate due to heterogeneity in cavity liner types, restorative materials, caries excavation techniques, and non-uniform outcome reporting. Additionally, variability in study quality introduced a risk of bias that could compromise pooled estimates.

#### Grading of evidence using GRADE

A critical appraisal of the included studies was performed to grade the overall body of evidence (Table 2).

##### Risk of bias

The included studies generally showed some limitations, primarily due to concerns about randomization procedures and allocation concealment. Operator blinding was not considered a limitation, as it is typically not feasible in restorative procedures. However, blinding of outcome assessors was inconsistently reported, which may have introduced some detection bias.

##### Imprecision

Several studies had small sample sizes and relatively short follow-up durations, reducing statistical power and increasing uncertainty, especially for postoperative hypersensitivity outcomes.

##### Inconsistency

There was moderate heterogeneity among the studies, mainly due to differences in cavity liner materials, restorative techniques, and clinical protocols. ‘Failure’ was downgraded, because most studies did not directly report this outcome and due to a lack of validated outcome measures. These variations contributed to differing success rates and sensitivity outcomes across studies.

##### Indirectness

The evidence was directly applicable to clinical practice, as the included studies evaluated cavity liners and no-liner approaches in patients with deep carious lesions in vital permanent teeth. There was no need for downgrading.

##### Publication bias

No strong evidence of publication bias was detected. However, the limited number of large, high-quality RCTs means that selective reporting cannot be completely ruled out.

##### Rating the body of evidence

Considering the issues with risk of bias, imprecision, and heterogeneity, the certainty of evidence was rated as low for tooth survival, secondary caries prevention, and restoration longevity, and very low for postoperative hypersensitivity.

## Discussion

This systematic review was commissioned by the EFCD, ESE, ORCA and DGZ with the revised question. ‘In patients with deep caries lesions in mature or immature permanent teeth associated with no symptoms or those of a reversible pulpitis (P), is a cavity lining of a certain material (I) as efficacious as other materials or no cavity lining (C), in terms of a combination of patient and clinical reported outcomes (O), with “failure” as the most critical outcome?’ This review addresses a key clinical question that has not been comprehensively evaluated: whether cavity liners improve outcomes in deep caries management. By synthesizing evidence across liner types and comparing their use to no liner, while applying risk of bias and GRADE assessments, this review offers an evidence-based perspective to guide selective rather than routine liner use in restorative practice.

The aggregated evidence from 12 randomized controlled trials indicates that the use of cavity liners, regardless of material, does not consistently improve clinical outcomes such as failure rate, pulp vitality, postoperative hypersensitivity or prevention of secondary caries. Six studies directly comparing lined to unlined restorations [30, 34–38] showed no significant benefit from using liners, especially in trials with low risk of bias, such as Singh et al. [35] and Banomyong et al. [30]. Still, the certainty of evidence for the outcomes “failure” and “tooth survival” were rated as very low and low and should be analysed carefully. CH and GIC showed no statistically significant differences in maintaining pulp vitality, though outcomes varied between studies. Neither material consistently reduced secondary caries or postoperative sensitivity (*p* > 0.05 in all relevant comparisons). Although newer materials labelled as “bioactive ionic liners”, such as TheraCal LC [31, 32] and Activa BioACTIVE [29] have shown favourable outcomes in isolated studies, the evidence remains limited and inconsistent. Based on GRADE assessment however, the certainty of evidence for “secondary caries prevention” was rated as low and for “postoperative hypersensitivity” as very low, further supporting cautious interpretation and underscoring that liner use should not be driven by expectations of POHS reduction alone.

More decisive for treatment success were the type of restorative material and the thickness of remaining dentin. Composite resins showed consistently high success rates with or without liners [29, 35], largely due to superior sealing and marginal adaptation. GICs, though frequently studied [41, 42], were limited by inferior mechanical properties in earlier formulations. Based on GRADE assessment, the certainty of evidence for “restoration longevity” was rated as low, due to study limitations and imprecision, suggesting that while composites appear to perform well, further high-quality, long-term studies are needed to confirm these trends across materials. While most of the 12 RCTs included in this review did not report exact dentin thickness in millimetres, several described cavity depths in relative terms, such as lesions reaching the inner quarter or involving more than two-thirds of dentin. These cases, managed with selective or stepwise excavation, consistently demonstrated high pulp vitality retention (≥ 94.6%) and low rates of postoperative sensitivity​. Supporting experimental research has shown that a remaining dentin thickness of 0.5 mm can reduce pulpal irritation by approximately 75%, and up to 90% with 1 mm, while 2 mm of hard dentin typically prevents pulpal response entirely​ [43, 44]. These findings suggest that preserving dentin is a more important factor for preserving pulp vitality than the use of additional materials such as liners.

Comparing these findings with formerly conducted systematic reviews, one can observe similar results. The systematic review and meta-analysis by Da Rosa et al. [1] evaluated the necessity of CH liners in deep caries lesions. Their findings suggested that CH liners do not improve clinical success rates in primary or permanent teeth, reinforcing the conclusions drawn from the current study. Da Rosa’s review included 17 studies, of which only two were in permanent teeth, and most RCTs had a high risk of bias due to inadequate randomization and short follow-up periods. Beiruti et al. [45] conducted a review on the effectiveness of resin-based and GIC sealants, emphasizing that while resin-based materials had better retention, their long-term impact on caries prevention was comparable to GIC-based alternatives. The included RCTs had mixed quality, with some studies showing high risk of bias due to inconsistent follow-up durations and methodology variations. Garcia-Mota et al. [46] also examined light-cured calcium silicate-based cements as pulp therapeutic agents. Their meta-analysis concluded that these materials had limited long-term performance in direct pulp capping but were reliable for indirect pulp capping, suggesting that while certain liner materials may have potential, their long-term benefits remain uncertain. The review included ten RCTs, some of which exhibited high risk of bias due to limited follow-up duration and small sample sizes. The Cochrane systematic review on cavity liners in Class I and II resin-based composite restorations by Schenkel and Veitz-Keenan [9] found inconsistent evidence regarding the effect of liners on POHS and no significant impact on restoration longevity, further reinforcing the notion that liners may not always be necessary. The included studies were mostly of low to moderate quality, with variability in study designs and inconsistent outcome reporting leading to uncertain conclusions, which further contributed to the decision not to conduct a meta-analysis.

Our findings support the notion that the use of cavity liners in deep caries management does not significantly improve clinical outcomes. While certain cavity liners might offer benefits in certain clinical scenarios, the overall success of restorative treatment relies on other factors. These include appropriate selection of restorative materials, minimally invasive caries removal techniques and the clinician’s ability to achieve proper sealing and adaptation. This aligns with emerging evidence that emphasizes operator technique and decision-making as key determinants of long-term treatment success.

### Limitations

This review highlighted several limitations of the existing clinical evidence, such as a high variability in study design, including differences in caries removal methods, liner materials, and outcome measures. Short follow-up periods (typically 12 months) limited the ability to assess long-term restoration success or secondary caries prevention. Finally, high risk of bias in some studies due to inadequate randomization, lack of blinding, and high loss to follow-up limits their scientific value.

### Clinical implications

The available clinical evidence does not support the regular use of cavity liners in deep caries management in permanent teeth with the aim of reducing failure, secondary caries or postoperative hypersensitivity. However, this lack of evidence does not necessarily mean that liners have no clinical benefit at all. Rather, their use should be tailored to specific clinical contexts. In cases of deep caries approaching the pulp, where remaining dentin thickness is minimal, the use of a liner may provide a an additional barrier to protect the pulp from mechanical or chemical damage. If so-called “bioactive” liners clinically outperform “conventional”, e.g. GIC liners, remains an open question.

## Conclusion

The findings of this systematic review, along with existing literature, suggest that cavity liners do not consistently provide superior clinical outcomes compared to no liners in deep caries management. Restoration success is primarily influenced by restoration material selection, caries removal technique and residual dentin thickness than by the application of a liner. Certain materials might have the potential to increase quality of life through reduction of postoperative hypersensitivity. Nonetheless, further research with standardized methodologies and longer follow-up durations is needed to refine clinical recommendations regarding cavity liner use.

## Data Availability

No datasets were generated or analysed during the current study.

## Appendix

**Table 4:** Extraction tables for included studies

Reference Study design	Participants	Drop-out Rate	Intervention	Control	Outcome(s)	Results	Remarks
**Baraka et al. 2022** RCT Egypt	*N* = 49 patients Age 6–9 y **Follow-up: 12 m** **Inclusion**: deep (D3), active caries lesions in first permanent molars, **open apex** **Exclusion**: irreversible pulpitis, spontaneous pain, pain on percussion, abscess, sinus, radiographic signs of irreversible pulp pathology, internal or external root resorption Baseline characteristics: ICDAS assessment, periapical radiographs ◊ selective caries removal Dentin thickness: Periapical x-ray to confirm lesion depth (inner third of dentin); Not explicitly reported differences between groups: none	49 participants, 108 restorations (36/36/36) **Loss to follow-up (overall): 36.1%**	**I1) SDF + KI** 38% SDF application followed by potassium iodide (KI) and RMGIC liner AND composite filling **I2) SDF** 38% SDF application and RMGIC liner AND composite filling	**C) RMGIC liner** AND composite filling	*primary outcome*: **secondary caries** **postoperative pain** (yes > 3weeks post-op) **tooth vitality** (thermal) **abscess/fistula** (clinical investigation) **restoration success** modified USPHS, Ryge: A = ideal B = clinically acceptable C = clinically unacceptable & replacement needed *secondary outcome*: **pulpal pathology** (periapical x-rays: baseline, 6 and 12 m)	69 restorations (25/22/22) • **100% secondary caries prevention (***p* **= 0.63)** • **minimal postoperative sensitivity with 96% pain-free outcomes (***p* **= 0.99)** • **preserved tooth vitality in all cases (***p* **= 0.99)**	Trial was pre-registered Randomization software Allocation concealment for investigator, patient and statistician X-ray analysis was assessed blindly High loss to follow-up (due to COVID) but evenly distributed among intervention groups. **Conclusion**: *No differences among the 3 groups regarding secondary caries prevention*,* pain*,* pulpal health. At 12 m RMGIC had significantly better clinical restoration color and luster than SDF and SDF + KI and better staining than SDF.*
**Efes et al. 2006** RCT Türkiye	*N* = 54 patients Age 18–48 y 25 m 29 f **Follow-up: 12 m** **Inclusion**: good oral hygiene, only molars, teeth in occlusion **Exclusion**: tooth mobility Baseline characteristics: Caries assessment using dental loupes and mirror, additional x-ray diagnosis ◊ non-selective caries removal Dentin thickness: Not reported differences between groups: none	54 participants, 108 restorations (27/27/27/27) **Loss to follow-up (overall)**: **7%**	**I1) Composite Liner** AND Ormocer **I2) Composite Liner** AND Nanofiller Composite	**C1) NO Liner** AND Ormocer **C2) No Liner** AND Nanofiller Composite	*primary outcome*: **USPHS modified Ryge criteria** A = ideal B = clinically acceptable C = clinically unacceptable & replacement needed D = fractured restoration *secondary outcome*: /	108 restorations (27/27/27/27) • **secondary caries prevention was 100% across all groups (***p* **> 0.05)** • **postoperative sensitivity was negligible, with 100% pain-free outcomes (***p* **> 0.05)**	No Randomization Allocation concealment for investigator& patient No post- operative X-ray analysis Low loss to follow-up **Conclusion**: *There was no statistically significant difference between the restoration with or without the application of flowable materials. There were no statistically significant differences detected among the materials.*
**Banomyong et al. 2011** RCT Thailand	*N* = 75 patients Age 18–40 y (Mean 22.8y) 18 m 57 f **Follow-up: 12 m** **Inclusion**: 1–4 moderate-to-deep, primary occlusal caries in first or second molars (no exposure of the pulp), no cares on other surfaces, no signs of pulpal and periapical disease, pre-operative sensitivity was relieved immediately after stimulus removal, at least one antagonist tooth with occlusal contact, healthy or mildly-inflamed gingival tissues, without gingival recession/alveolar bone loss **Exclusion**: psychological disorders, Neurological diseases, Temporo-mandibular disorders, Pregnancy or breast feeding, regular use of analgesic or anti-inflammatory drugs, allergy to materials, previous restoration(s) and/or tooth surface loss (attrition, erosion, abrasion, or abfraction), ‘‘cracked tooth syndrome’’, orthodontic treatment within the previous 3 m Dentin thickness: after caries removal cavity depth measured with periodontal probe, exact residual thickness not provided **Baseline characteristics**: clinical examination and pre-operative x-ray (BW). Caries detector dye (KURARAY) ◊ selective caries removal differences between groups: none	75 participants, 110 restorations (26/30/26/28) **Loss to follow-up (overall): 25%**	**I1) GIC lining** AND two-step total-etching adhesive **I2) GIC lining** AND two-step self-etching adhesive ALL WITH nano-filled resin composite	**C1) No GIC lining** AND two-step total-etching adhesive **C2) NO GIC lining** AND two-step self-etching adhesive ALL WITH nano-filled resin composite	*primary outcome*: **Clinical Criteria**: Patient Satisfaction Fracture and Retention Marginal Adaptation Recurrent Caries Post-operative Sensitivity (Score 1–5) 1: Clinically excellent 2: Clinically good 3: Clinically satisfactory 4: Clinically unsatisfactory 5: Clinically poor *secondary outcome*: /	83 restorations (23/21/19/20) • **secondary caries prevention and postoperative sensitivity were excellent across all groups, with success rates of 98.8%.** • **Patient satisfaction was also high (***p* **> 0.05)**	Sample Size Calculation (power of 0.9) ◊ 26 restorations per group Randomisation not described! Allocation concealment unclear! Investigator blinded No postoperative radiographic assessment **Conclusion**: *Moderate-to-deep occlusal cavities might be restored successfully with a nanofilled resin composite bonded with the two-step adhesives*,* regardless of whether a GIC liner is placed or not.* *In the short term*,* the GIC lining did not affect the quality of an occluso-posterior resin composite restoration with surrounding enamel margins.*
**Torres et al. 2020** RCT Brazil	*N* = 30 patients Mean Age 38 y 20 m 40 f **Follow-up: 24 m** **Inclusion**: deep caries lesions (ESE definitions)/deep defective restorations reaching inner quarter of dentin, with a zone of hard dentin between cavity and pulp (radiographic), two posterior permanent teeth in each patient, vital pulp, absence of painful symptom, no history of hypersensitivity, good oral health **Exclusion**: extremely deep caries (ESE definition), penetrating entire thickness of dentin, unrestorable crown, mechanical pulp exposure during caries removal, teeth previously treated with direct pulp capping, teeth exhibiting spontaneous/constant pain, heightened or lingering response to thermal pulp testing/symptoms of irreversible pulpitis, severe systematic disease, allergies to dental composites, parafunctional habits and bruxism, periodontal disease **Baseline characteristics**: sensitivity test to cold stimulus, crown percussion and palpation of soft tissue on periapex, radiographic analysis, periodontal probing and mobility test ◊ selective caries removal Dentin thickness: Not explicitly reported; pre-operative x-ray confirmed lesion in inner quarter of dentin, but with firm layer of dentin remaining differences between groups: none	30 participants, 60 restorations (30/30) **Loss to follow-up (overall): 16.67%**	**I) Light cured glass-ionomer composite liner** AND Universal dual-cure self-etching adhesive AND Ormocer nanohybrid composite	**C) NO Liner** Universal dual-cure self-etching adhesive AND Ormocer nanohybrid composite	*primary outcome*: **Clinical performance (FDI criteria)** (Score 1–4) (1) Clinically excellent/very good (2) Clinically good (3) clinically sufficient/satisfactory (4) clinically unsatisfactory *secondary outcome*: **Pulpal Health** cold Testing, percussion, palpation and radiographic analysis	60 restorations (30/30), two restorations per patient • **secondary caries prevention remained 100% (***p* **> 0.05)** • **marginal adaptation was clinically excellent in 83.33% of cases (***p* **> 0.05)** • **no postoperative sensitivity in any group (***p* **> 0.05)**	Trial was pre-registered Sample Size Calculation (power of 0.8) ◊ 30 restorations per group Randomization software rubber dam Blinding of patient and examiner, operator not blinded Drop-out rate low! Sensitivity analysis to evaluate influence of drop-out on results No radiographic analysis **Conclusions**: *The application of a light-curing glass-ionomer composite liner did not influence the clinical performance of deep restorations with bulk-fill Ormocer composite.*
**Ahmed et al. 2024** RCT Pakistan	*N* = 20 patients Age 25 y 11 m 9 f **Follow-up: 36 m** **Inclusion**: 2 or more deep caries (ICDAS 5/6) one on each side of the mouth, symptomless and vital teeth, no sign of pulpitis or pathological lesions, good oral hygiene, good likelihood of recall availability **Exclusion**: adverse medical history, allergies or systematic diseases, pregnant or lactating females, visibly cracked teeth, sensitivity to percussion, severe chronic periodontal problems **Baseline characteristics**: preoperative photographs, bitewing radiographs for caries assessment, pulp vitality via cold test ◊ selective caries removal Dentin thickness: Not explicitly reported; after caries excavation cavity depth evaluated with Prepometer differences between groups: none	40 restorations (20/20) **Loss to follow-up (overall): 5%**	**(1) Bioactive ionic glass- ionomer liner** **AND** **nanofilled resin composite**	**(2) Resin modified glass- ionomer liner (etch-and-rinse mode)** **AND** **nanofilled resin composite**	*primary outcome*: **Clinical success using FDI criteria**: (1) Clinically very good (2) Clinically good (3) Clinically sufficient/satisfactory (4) clinically unsatisfactory (5) Clinically poor *secondary outcome*: /	40 restorations at baseline (20/20) 38 restorations at 6 m (19/19) • **all groups had similarly high clinical success rates (94.7%) with no differences in pulp vitality or postoperative sensitivity**	Trial was pre-registered Sample Size calculation (power 0.8) ◊ 40 restorations Randomization with coin flip Patients and Assessor blinded Two independent assessors Low Loss to follow-up pre-operative x-ray rubber dam **Conclusion**: *Resin composite restorations showed acceptable clinical performance over 3 years either lined with bioactive ionic or resin-modified glass- ionomer liners after selective caries excavation preserving pulp vitality.*
**Banomyong and Messer 2013** RCT Thailand	*N* = 53 patients Age 18–30 y 22 m 31 f **Follow-up: 24 m** **Inclusion**: deep occlusal caries, first or second upper/lower permanent molar without other defects, at least one opposing tooth, healthy periodontal tissues/mildly inflamed **Exclusion**: medical problems, orofacial pain, pulpal or periapical disease **Baseline characteristics**: preoperative hypersensitivity (but relieved through intervention), spontaneous pain, caries detector dye ◊ non-selective caries removal Dentin thickness: After Caries removal cavity depth (proxy for dentin thickness) measured using using periodontal probe; exact residual thickness not provided differences between groups: none	53 participants, 81 restorations (44/37) After 1 and 2 y: 62 restorations (31/31) **Loss to follow-up (overall): 36%**	**I) GIC lining** (0.5–1 mm thickness over entire dentin surface) AND two-step total-etching adhesive or two-step self-etching adhesive, nano-filled resin composite	**C) No GIC lining** AND two-step total-etching adhesive or two-step self-etching adhesive, nano-filled resin composite	*primary outcome*: **postoperative hypersensitivity** Objective signs Subjective symptoms No response to cold or electric pulp testing (assessed a “present” or “absent”, scale 0-100) *secondary outcome*: /	62 restorations (31/31) • **No hypersensitivity among all groups at all follow-ups**	Sample Size Calculation (power of 0.9) ◊ 12 restorations per group No rubberdam isolation with every restoration No postoperative radiographic assessment HIgh loss to follow-up **Conclusion**: *In conclusion*,* the absence of GIC lining did not increase the risk of postoperative hypersensitivity or pulpal complications in deep occlusal cavities restored with resin-based restorations at the 2-year evaluation.*
**Pereira et al. 2017** RCT Brazil	*N* = 98 patients Age 15-30y (mean 23.3y) 43% m 57% f **Follow-up: 13 m** **Inclusion**: deep carious lesions (inner third of dentin) in permanent molars and premolars **Exclusion**: periapical or periodontal lesions, necessity of extensive indirect restorations, any diagnosis of pulp alteration (cold testing), root exposure, or non-carious cervical lesion, hypersensitivity and pulp exposure during caries removal **Baseline characteristics**: bite-wing x-ray, measured dentin consistency and colour, as well as dentin moisture ◊ stepwise caries removal Dentin Thickness: ≥2/3 of dentin retained; measured using x-ray software differences between groups: none	98 Recruited participants 98 restorations (49/49) **Loss to follow-up (overall): 20%**	**I) Calcium Hydroxide Liner** AND Resin-modified GIC (no etching)	**C) No Liner** AND Resin-modified GIC (with dentin etching)	*primary outcome*: **Response to Cold Sensitivity Test** Risk of negative response (95% CI) **Clinical Aspects of Dentin** Change in colour scores, median (1st/3rd quartiles)/Scores from 1 to 5 Change in consistency scores, median (1st/3rd quartiles)/Score from 1 to 4 Moisture, ratio of dry dentin (95% CI) **Radiographic Analysis** Change in thickness of remaining dentin, millimetre mean (SD) *secondary outcome*: /	98 restorations (49/49) • **pulp vitality success rates were 98.6% in both groups (***p* **> 0.05)** • **No significant differences were found in postoperative sensitivity or dentin characteristics (***p* **= 0.402)**	Trial was pre-registered Sample Size calculation (power to 0.8) ◊49 teeth per group Computer based randomization Rubber dam post-operative x-ray Low loss to follow-up **Conclusions**: *On the basis of this 3-month clinical trial*,* the additional application of calcium hydroxide liners does not appear to provide any additional benefit to provisional restoration during stepwise excavation.*
**Singh et al. 2019** RCT India	*N* = 198 patients Age 14–54 y (Mean 23.44y) 116 m 82 f **Follow-up: 12 m** **Inclusion**: least 12 years old, permanent mandibular first and second molars exhibiting primary deep occlusal and occlusal-proximal caries lesion involving at least two-thirds of dentin (detected radiographically), positive response to cold test and electric pulp test, absence of spontaneous pain, negative sensitivity to percussion, absence of mobility, sinus, fistula **Exclusion**: immunocompromised status/debilitating systemic diseases, severe periodontal disease/rampant caries, cusp loss, periapical/interradicular radiolucency/any other radiographic findings indicative of pulp necrosis **Baseline characteristics**: depth assessment using x-rays in case of occlusal lesions ◊ selective caries removal Dentin thickness: radiographic assessment before caries excavation, Inner 1/4 of dentin retained differences between groups: none	198 participants, 198 restorations (66/66/66) **Loss to follow-up (overall): 11.11%**	**I1) Calcium hydroxide liner** AND Composite restoration (ER adhesive) **I2) Resin-modified GIC Liner** (pulpal and axial walls) AND Composite restoration (ER adhesive)	**C) No Liner** AND Composite restoration (ER adhesive)	*primary outcome*: **Pulpal Health outcome** **Success**: pulp sensitivity to cold and electric test and absence of periapical/interradicular alterations on x-rays (combined outcome). **Failure**: spontaneous pain, fistula, swelling, mobility (clinical). Radiolucency on apex or furcation are and emergence of internal or external pathological resorption (radiographic) *secondary outcome*: /	176 restorations (63/57/56) • **Pulp vitality success rates were 96.8%, 96.5%, and 94.6%, respectively (***p* **= 0.71)** • **secondary caries prevention was 100% across all groups** **Overall failures**: 7 (2 = irreversible pulpitis, 5 = pulpal necrosis and radiographic changes) 3 failures in Group I1); 2 in group I2); 2 in group C)	Trial was pre-registered Sample Size Calculation (power to 0.9) ◊65 teeth per group Rubber dam Blinding of patient and examiner, operator not blinded post-operative x-rays **Conclusions**: *It can be concluded that partial caries excavation has a high success rate to treat deep carious lesions in permanent teeth after 12 months of follow-up*,* indicating that the retention of carious dentin does not interfere with pulp vitality. Also*,* the success of the treatment is independent of the lining material used over the demineralized dentin.*
**Hodosh et al. 1991** RCT USA	*N* = 80 patients Age > 18 y ? m ? f **Follow-up: 2 y** **Inclusion**: deep carious lesions requiring amalgam restorations deep caries = depth of decay with remaining dentin wall thickness =/< 1 mm **Exclusion**: exposed pulp, periapical pathosis (clinically or radiographically), elevated temperature and/or white blood cell count, malaise, allergy to potassium nitrate and/or zinc polyacrylate, teeth with pulsating pain, systemic steroids, antibiotics, antihistamines and topical potassium nitrate toothpaste usage **Baseline characteristics**: pre-operative x-ray ◊ non-selective caries removal Dentin thickness: Clinical observation and estimated to be ≤ 1 mm differences between groups: none	80 restorations (42/40) **Drop-out rate not mentioned**	**I) Potassium nitrate-polycarboxylate cement Liner** (KNO_3_ + PCA) AND Amalgam	**C) Polycarboxylate cement Liner** (PCA) AND Amalgam	*primary outcome*: **Periapical pathosis** **Pulp vitality** **Postoperative sensitivity** *secondary outcome*: /	80 restorations (42/38) • **potassium nitrate liners significantly reduced postoperative sensitivity (95.2% vs. 78.9%,** *p* **< 0.01), preserved pulp vitality (100% vs. 81.6%,** *p* **< 0.05)**** and reduced periapical pathosis (0% vs. 18.4%,** *p* **< 0.05)**	No Sample Size Calculation Randomization (flip of coin) Blinding of both patient AND operator! Post-operative x-ray analysis **Conclusions**: *The results of this study indicated that potassium nitrate-polycarboxylate cement was an effective agent for preserving pulp vitality. It apparently helped prevent periapical pathoses from forming under amalgam restorations*,* while it diminished postoperative pain.*
**Bayoumy et al. 2021** RCT Saudi Arabia	*N* = 50 patients Age? ? m ? f **Follow-up: 12 m** **Inclusion**: one posterior permanent tooth with occlusal primary deep caries (radiographically extending into inner 1/3 of dentin and no connection to pulp), max of two teeth per patient **Exclusion**: teeth with restorations, spontaneous pain or prolonged pain after cold test, allergy to restorative materials, teeth mobility (periodontal disease or trauma), gingiva inflammation, external or internal resorption with adverse pulpal reactions, cervical caries, severe wear (parafuncion) **Baseline characteristics**: caries risk assessment, pulp cold test, percussion test, clinical signs of inflammation, periapical radiographs, pain assessment using VAS ◊ selective caries removal Dentin thickness: No reported differences between groups: none	50 restorations (25/25) **Loss to follow-up (overall): 18%**	**I) TheraCal** Resin-modified calcium silicate (1 mm incremental layer, light-cured) AND Temp. restoration: GIC	**C) Dycal** Calcium hydroxide (thickness of 0.8–1 mm) AND Temp. restoration: GIC	*primary outcome*: **postoperative pain** (modified VAS scale): 0 = no pain (0) 1–3 = mild pain (1) 4–6 = moderate pain (2) 7–10 = severe pain (3) *secondary outcome*: bacterial count (omitted, not relevant)	41 restorations (20/21) • **TheraCal liners led to significantly higher pain-free rates at 12 months (100% vs. 81%,** *p* **< 0.05)** • **restoration success rates were similarly high (94.7% vs. 89.5%,** *p* **> 0.05)**	Trial was pre-registered Sample Size calculation (power 0.8) ◊ 50 restorations Randomization programme Patients and Assessor blinded Low Loss to follow-up Pre-operative x-ray Rubber dam **Conclusion**: *Resin-modified calcium silicate and calcium hydroxide presented clinically effective pulp capping materials through relieving pain. The results indicated that resin-modified calcium silicate was more effective in reducing pain than that of calcium hydroxide as indirect pulp capping agent.*
**Gürcan and Seymen 2019** RCT Türkiye	*N* = 95 patients Age 4–15 y, Mean 8.55y 43 m 52 f **Follow-up: 24 m** **Inclusion**: secondary and first premolars from healthy and cooperative children with nonclinical and radiographic evidence of infection, with indications for indirect pulp treatment **Exclusion**: teeth with clinical abscess, signs of inflammation (abnormal mobility, tenderness to percussion and palpation, spontaneous pain), absence of vitality (thermal pulp test), progression of caries to pulp, perforation of pulp, intermittent or irregular lamina dura, expanded range of periodontal ligament, periapical and furcation radiolucency, internal or external resorption **Baseline characteristics**: Panoramic X-rays, ice-cold tests ◊ selective caries removal Dentin thickness: Not reported differences between groups: none	295 restorations (91/89/115) **Loss to follow-up (overall): 5.1%**	**I) Dycal** AND Composite Restoration	**C1) ProRoot MTA** **C2) TheraCal LC** AND Composite Restoration	*primary outcome*: **Clinical and Radiographic Assessment** **Restoration Success using modified USPHS criteria** (pain, fistulas, pain upon percussion, pathological mobility, abscess, vitality symptoms) *secondary outcome*: /	295 restorations (91/89/115) • **success rates of 94.4% for ProRoot MTA, 87.8% for TheraCal, and 84.6% for Dycal, with no significant differences in clinical or radiographic outcomes (***p* **> 0.05)** • **No statistical differences between material groups on radiological pathologies (***p* **> 0.05)**	Sample Size Calculation (power to 0.8) ◊ 92 restorations Rubber dam Post-operative x-rays Low loss to follow-up **Conclusions**: *Treatment success was independent of capping material and was also compatible with other study results that indicate the importance of the hermetic seal of the restoration. No statistically significant differences were found between the results comparing the materials according to the modified USPHS criteria (p > 0.05). These results support the idea that the success of IPC (indirect pulp capping) is independent from the capping material.*
**Hashem et al. 2015** RCT UK	*N* = 53 patients Age 18–76 y 32 m 21 f **Follow-up: 12 months** **Inclusion**: 18 y or above, in good general health, at least one deep carious lesion extending ≥¾ into dentine (confirmed radiographically), clinical symptoms of reversible pulpitis, positive pulp response to electric or thermal tests, no periapical (PA) changes visible on baseline radiographs **Exclusion**: Clinical signs of irreversible pulpitis (e.g., spontaneous pain, lingering pain), presence of fistulas, abscesses, or facial swelling, tooth mobility or tenderness to percussion, anterior teeth where aesthetics would be compromised, pregnant women (due to radiographic exposure) **Baseline characteristics**: Positive response to both electric pulp test and cold stimulation, no visible PA lesions on initial PA radiographs ◊step-wise selective caries removal Dentin thickness: Not explicitly reported; pre-operative radiographic assessment: lesions extended > 3/4 into dentin differences between groups: Teeth with baseline severe symptoms of reversible pulpitis received more GIC restorations compared with Biodentine as symptom intensity was not considered during the material randomization	72 restorations (36/36) **Loss to follow-up (overall): 8.3%**	**I) Biodentine** AND Composite Restoration	**C) Fuji IX GIC** AND Composite Restoration	*Primary outcome*: **Tooth vitality (cold/EPT test)** **Symptoms (pain**,** swelling**,** mobility)** **Periapical radiolucency on x-ray/CBCT** *Secondary*: /	72 restorations (36/36) • **Clinical success (vitality) equal: 83.3% for both groups** • **No significant difference in failure rate (***p* **= 0.91)** • **Biodentine associated with more healed periapical lesions: 71% of healed lesions received Biodentine vs. 88% of new/progressed lesions received Fuji IX Significant (***p* **= 0.02)**	Sample Size Calculation (power to 0.8) ◊ 72 restorations Randomization stratified by cavity size Low Loss to follow-up **Conclusions**: *The null hypothesis stating there is no difference in the dentine-pulp response between Biodentine and Fuji IX clinically was accepted but not radiographically.*

**Table 5:** Effectiveness of different cavity liners compared to no cavity lining in managing deep caries in vital permanent teeth

Search Strategy:		
Nr.	PICOTS	Search terms
1	P	Caries OR Carious lesion OR Tooth decay MeSH: Dental caries
2	P	Permanent teeth OR Secondary dentition OR Adult teeth MeSH: Adult dentition OR Dentition, adult OR Dentition, permanent OR Secondary dentition
3	I / C	Cavity lining OR Cavity base OR Cavity sealing OR Calcium hydroxide OR Hydraulic calcium silicate cement OR Glass ionomer cement OR GIC MeSH: Dental cavity Lining OR Calcium hydroxide OR Glass ionomer cement OR Silicate cement
4	excluded	Trauma AND Root
5	S	Filters: published after 1990; written in English
6		#1 AND #2 AND #3 NOT #4
